# Vitamin D Levels in Patients with Active and Remission Graves’ Disease

**DOI:** 10.3390/medicines10070041

**Published:** 2023-07-06

**Authors:** Natapon Rattanamusik, Suriyon Uitrakul, Atchara Charoenpiriya

**Affiliations:** 1 Department of Medicine, Maharaj Nakhon Si Thammarat Hospital, Nakhon Si Thammarat 80000, Thailand; phingsmart@gmail.com (N.R.); fong109@gmail.com (A.C.); 2 Department of Pharmaceutical Care, School of Pharmacy, Walailak University, Nakhon Si Thammarat 80160, Thailand

**Keywords:** vitamin D, calcitriol, Graves’ disease, hyperthyroidism

## Abstract

Background: The association between Graves’ disease (GD) and serum vitamin D levels has been studied for decades although the results were controversial. Moreover, the difference in vitamin D levels between the different stages of GD is not well studied. Therefore, this study aimed to compare the vitamin D levels between active and remission GD and to investigate the factors affecting vitamin D levels in GD patients. Methods: This cross-sectional study was performed between 1 January to 31 December 2021. The eligible patients were in either the active or remission stage of GD. The demographic and clinical data of the patients willing to participate in the study were collected, as well as their vitamin D levels. Comparisons of continuous parameters between the active and remission groups were performed using the Mann–Whitney U test, while categorical parameters were performed using the Chi-square test. Results: 75 patients were diagnosed with GD, with 54.7% in the active stage. The mean vitamin D level was lower in the active GD group than in the remission GD group (28.23 vs. 31.58 ng/mL, respectively, *p*-value 0.079). The prevalence of vitamin D deficiency (i.e., serum vitamin D level < 20 ng/mL) in the active GD group was 14.6%, and in the remission GD group was 0% (*p*-value 0.02). Moreover, there was a significant negative correlation between the serum vitamin D level and serum free T4 level (*p*-value 0.03). Conclusions: In spite of non-significance, patients with active GD had lower mean vitamin D levels compared to those with remission GD. The prevalence of vitamin D deficiency was significantly higher in the active GD patients. Additionally, a negative correlation between serum vitamin D levels and serum free T4 levels was observed in this study.

## 1. Introduction

Graves’ disease (GD) is the most common cause of hyperthyroidism in clinical practice, which involves several etiological factors, including genetic and environmental factors (e.g., iodine level, stress, infection, and smoking). Thyrotropin-receptor antibodies (TRAb) at thyroid and fibroblast orbital cells are the main mechanism of pathogenesis to induce hypertrophy and hyperplasia of the thyroid gland [1,2]. TRAb causes inflammation and differentiation of the fibroblasts and turns them into myofibroblasts and adipose tissues. Patients with enlarged and diffusely overactive thyroid glands have a variety of symptoms, such as weight loss, palpitation, heat intolerance, fatigue, tremor, and ocular abnormalities, including exophthalmos, lid lag, and lid retraction [2,3].

Calcitriol (1,25-dihydroxy vitamin D or 1,25(OH)2D) is associated with calcium homeostasis and nucleus differentiation in the human body [4]. In addition, calcitriol directly inhibits dendritic cells and CD4+ helper T lymphocyte cells via binding at the major histocompatibility complex (MHC) class II [5]. Consequently, serum vitamin D levels can affect the prognosis of patients with GD. Although the association between GD and vitamin D levels has been studied for decades, it is still unclear whether vitamin D deficiency is related to the pathogenesis or the consequence of autoimmune thyroid diseases [6]. Several studies reported lower vitamin D levels in patients with GD than in the normal population. For instance, a study in Korea reported that the prevalence of calcidiol deficiency in patients with Hashimoto thyroiditis and Graves’ disease was higher than in the normal population [7]. In addition, a meta-analysis study reported a significantly lower mean vitamin D level and a lower odd ratio of vitamin D deficiency in the patients with GD compared to the non-GD population [8]. However, to the best of our knowledge, no study reports the relationship between vitamin D and thyroid function in Thai patients.

Thailand is a country in southeast Asia, which is exposed to a higher intensity of sunlight than other countries such as Korea and Japan. Despite the fact that Thai people are exposed to sunlight daily for a long time, the percentages of Thai people who had serum vitamin D levels less than 20 ng/mL and 50 ng/mL were 5.7% and 45.2%, respectively [9]. Moreover, the incidence of patients with GD in Thailand was 26.57 per 100,000 per year, so there was a high risk that the Thai GD patients would have vitamin D deficiency. Therefore, this study aimed to investigate serum vitamin D levels in patients with GD, as well as the associated factors of the level of vitamin D in such patients.

## 2. Materials and Methods

### 2.1. Patient Population

This cross-sectional study was performed at Maharaj Nakhon Si Thammarat Hospital, a tertiary hospital in southern Thailand, between January 2020 and December 2020. The inclusion criteria of the study were patients aged over 18 years, being diagnosed with GD, and being treated at Maharaj Nakhon Si Thammarat Hospital during the study period. Patients were diagnosed with GD based on the following criteria: diffusely enlarged goiter, Graves’ ophthalmopathy or dermopathy, low level of serum thyroid stimulating hormone (TSH) (<0.4 mIU/L), high level of serum free T4 (>1.8 pg/mL), and positive serum TRAb.

The patients with any conditions that affected vitamin D levels were excluded, including chronic kidney disease (CKD), cirrhosis, pregnancy, and on vitamin D supplements, as well as the known case of vitamin D deficiency.

Prior to the recruitment to this study, all patients were treated with either propylthiouracil (PTU) or methimazole (MMI), depending on their clinical presentation. In cases where the patients had severe disease, they may have received radioactive iodine solution based on the physician’s decision, resulting in, sometimes, the symptoms of hypothyroidism instead. These patients received levothyroxine for the management of their secondary hypothyroidism.

### 2.2. Study Design

The recruited patients were asked for their permission to participate in this study. Their consent to the study did not affect the quality of the treatment in any aspect. After their consent, physical examinations were performed on the patients, and their clinical data were collected from the patient medication records. The patient examination included signs and symptoms of hyperthyroidism, as well as thyroid size. Their blood samples were collected and sent to the laboratory for a thyroid function test, vitamin D2 levels, intact parathyroid hormone, calcium levels, and albumin levels. Other demographic data were collected during the physical examinations by doctors.

Later, the patients were divided into two groups based on the clinical stage of GD at the recruitment time: active and remission diseases. The active disease was defined as a patient who had high levels of free T3 and/or free T4 levels together with a low level of TSH at the time of the vitamin D measurement. On the other hand, remission disease was defined as a patient who has had normal thyroid function results for longer than six months (according to the medication records) until the time of vitamin D measurement. Both active and remission groups received the appropriate treatment depending on their symptoms.

### 2.3. Variables and Outcomes

The patient demographic data were collected from the patient report, medication records, and physical examination, including sex, age, weight, height, body mass index, goiter size, smoking status, medication of treatment (drug name and dose), and daily sun exposure time. Routine laboratory tests of thyroid function, i.e., intact parathyroid hormone (iPTH), free T3 level, free T4 level, TSH level, calcium, and albumin, were performed by the hospital laboratory. Serum vitamin D levels were measured using the electrochemiluminescence immunoassay (ECLIA) method with Cobas e801 machine (Roche Co.) from the hospital laboratory.

The primary outcomes of this study were the level of serum vitamin D and the prevalence of vitamin D deficiency in the patients with different stages of Graves’ disease. Vitamin D deficiency was defined as a vitamin D level of less than 20 ng/mL. The secondary endpoint was to evaluate the association between the stage of GD and vitamin D level, as well as the correlation of the vitamin D level with biochemical parameters in GD.

### 2.4. Statistical Analysis

The demographic and clinical data of the patients were described using descriptive statistics; continuous variables were expressed as mean with a 95% confidence interval (95% CI), and categorical variables were presented as numbers with percentages. The differences in the continuous and categorical variables between the two groups were analyzed using the Mann–Whitney U test and the Chi-square test, respectively. The Spearman correlation analysis was used to determine the factors associated with vitamin D levels. All statistical analyses were performed using IBM SPSS Statistics software version 28.0.0.0 (190). The *p*-values in this study were two-sided, and statistical significance was defined as a *p*-value < 0.05.

## 3. Results

### 3.1. Patient Characteristics

During the study period, 75 patients were recruited to the study, divided into 41 patients with active GD (54.7%) and 34 patients with remission GD (45.3%). In addition, of the 34 patients in the remission group, there were 6 patients who used methimazole as an antithyroid medication. The demographic data of the patients are shown in Table 1. The proportion of male patients was similar between the active and remission groups (31.7% and 23.5%, respectively). The mean age and mean BMI among them were not statistically significant. Moreover, there was no significant difference between the patients with active GD and remission GD in the mean sun exposure length (3.53 h/day vs. 3.08 h/day, respectively), the mean iPTH levels (45.81 pg/mL vs. 38.97 pg/mL, respectively), and the mean calcium levels (9.25 mg/dL vs. 9.35 mg/dL, respectively).

As the patients in the two groups were at different stages of GD, the characteristics of thyroid function were different. Patients with active GD had a shorter mean disease duration (834 days, 95% CI 527–1142 days) than those with remission GD (1454 days, 95% CI 1086–1822 days, *p*-value 0.003). The goiter sizes of both groups were significantly different (55.00 g vs. 31.56 g, respectively, *p*-value < 0.001). In addition, there were significant differences in the free T3 levels, free T4 levels, and TSH levels between the two groups.

### 3.2. The Serum Vitamin D Levels

**Figure 1:**
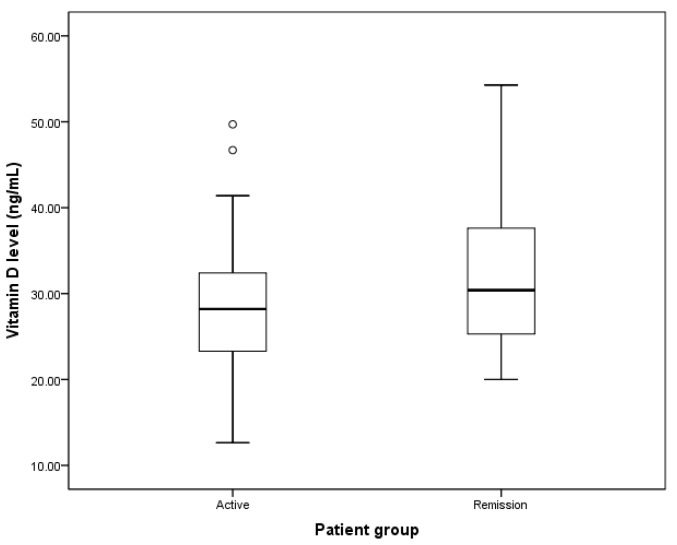
Mean vitamin D2 levels of the patients with Graves’ disease in the active and remission stages. White circles indicate the outliners.

Figure 1 shows the mean level of vitamin D in the patients in this study. The patients with active GD had an average vitamin D level of 28.23 ng/mL (95% CI 25.64–30.82 ng/mL), and the patients with remission GD had an average vitamin D level of 31.58 ng/mL (95% CI 28.80–34.37 ng/mL). However, there was no significant difference between the two groups (*p*-value 0.120).

**Figure 2:**
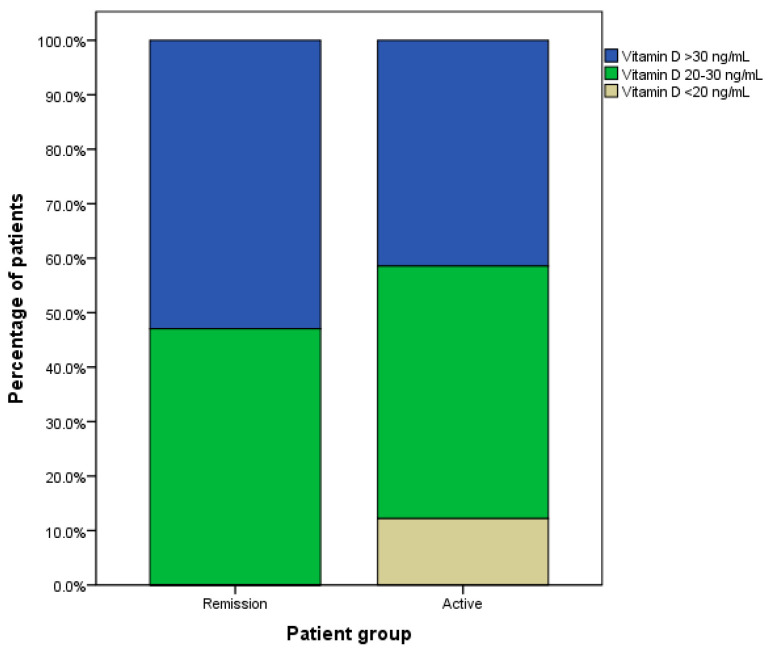
Percentage of Graves’ disease patients with different levels of vitamin D.

Based on the classification of vitamin D deficiency in this study, there were six patients (14.6%) in the active GD group who had vitamin D deficiency (Figure 2). At the same time, there was no patient with vitamin D deficiency in the remission GD group. The Chi-square test showed a significance between these percentages (*p*-value 0.020). The percentages of patients with serum vitamin D levels of 20–30 ng/mL were similar (44% of active GD and 47% of the remission GD). Still, the percentage of patients with serum vitamin D levels of more than 30 ng/mL were lower in the active GD group (42% of active GD and 53% of the remission GD).

### 3.3. The Factors Affecting Vitamin D Level

The association between serum vitamin D levels and thyroid-related tests is shown in Table 2. Only free T4 levels significantly negatively correlated with the patient vitamin D level (*p*-value 0.031). However, free T3 and TSH levels were not associated with the vitamin D levels of the patients. Moreover, iPTH and calcium levels were not related to vitamin D levels; neither was the daily sun exposure time of the patients.

Regarding other relevant parameters, the levels of free T3 and free T4 negatively correlated with TSH levels (r = −0.717 and r = −0.594, both *p*-values < 0.001), and free T3 levels strongly correlated with free T4 levels (r = 0.727, *p*-value < 0.001). Moreover, iPTH levels negatively correlated with serum calcium levels (r = 0.281, *p*-value 0.015).

## 4. Discussion

This observational study indicated that the mean level of vitamin D in the patients with active GD was lower than the patients with remission GD, although there was no statistically significant difference detected. Additionally, the percentage of patients with vitamin D deficiency in the active GD group was significantly higher than in the remission group. This was in accordance with a study in Japan that indicated a significantly lower calcidiol level in the patients with active GD than in the patients with remission GD and normal populations [10]. Moreover, this study showed a significant correlation between the free T4 level and vitamin D deficiency.

The association between GD and vitamin D has been studied for decades. However, there is still a high variation in the results. For example, an epidemiological study showed that the prevalence of vitamin D deficiency (<20 ng/mL) among the general Thai population was 5.7%, with a mean vitamin D level of 31.72 ng/mL [9]. For the people in southern Thailand (i.e., the location of this study), the prevalence of vitamin D deficiency was higher than the average of the country (7.4%), probably because this area has fewer hours of sunlight per day.

Regarding the patients with GD, to the best of our knowledge, the prevalence of vitamin D deficiency in such patients is not well studied in Thailand. In Hungary, a study reported the prevalence of vitamin D deficiency among GD patients at 64% compared to 30% of the healthy population [11]. In Japan, 40% of the female and 18% of the male GD patients were classified as having vitamin D deficiency [12]. Another study in Sweden indicated that approximately 46% of GD patients had vitamin D deficiency [13]. Those above-mentioned studies reported a much higher prevalence of vitamin D deficiency in GD patients than the present study (i.e., 8%), and the areas of study were most likely to contribute to this difference. Overall, meta-analysis studies indicated that patients with GD had 2.24 to 3.50 times more than normal people to develop vitamin D deficiency [8,14].

Graves’ disease not only affects the prevalence of vitamin D deficiency, but it also associates with the serum level of vitamin D. According to a study in Sweden, the average level of vitamin D in GD patients was 22 ng/mL, compared to 35 ng/mL in healthy controls [13]. However, the results from meta-analysis studies were still controversial. A meta-analysis study showed a significantly lower serum vitamin D level in GD patients compared to controls (0.77 ng/mL, 95%CI 0.42–1.12) [14], and another study reported 1.04 ng/mL (95% CI 0.57–1.52) of lower serum vitamin D levels in GD patients [8]. On the other hand, a meta-analysis showed 4.14 ng/mL lower serum vitamin D levels in GD patients, but this was not statistically significant [15].

Even though several studies showed the effects of GD on vitamin D levels, the difference between the active and remission stages of GD is not well reported. The only study that compared this difference was the study by Yasuda et al. [10]; it indicated a significantly lower serum vitamin D level in the patients without remission GD than those with remission GD (14.5 vs. 18.2 ng/mL, respectively). Similarly, the current study reported lower average vitamin D in patients with active GD than remission GD (28.23 vs. 31.58 ng/mL, respectively) although the difference was not statistically significant. In terms of the proportion of the patients with vitamin D deficiency, this study showed a significantly higher number of the GD patients with vitamin D deficiency in the active group compared to the remission group. Unfortunately, the study by Yasuda et al. [10] did not provide the number of the patients with vitamin D deficiency in the active and remission groups. Nevertheless, the results could be assumed that there were more patients with vitamin D deficiency in the non-remission group than the remission group.

The mechanism of association between vitamin D levels and thyroid hormones, as well as the stage of GD, is not well studied. One of the hypotheses was the specific chemokines of GD, such as IL-2, IFN-γ, and CXCL10; several studies suggested that calcitriol could regulate some immunomodulators, and malfunctioned immunomodulators resulted to more severity of GD. On the other hand, a decrease in serum vitamin D levels might result in higher inflammatory cytokines and then worsen the stage of GD [5]. Another hypothesis was malabsorption of calcium in active GD patients, together with an increase in vitamin D clearance; consequently, the serum vitamin D level in such patients was lower than the remission GD patients [16].

The results of this study highlighted the negative relation between free T4 levels and serum vitamin D levels; the patients with high free T4 levels tended to have low vitamin D levels. These findings supported the theory that active GD patients should have higher levels of free T4 than those with remission GD. In contrast, the study by Yasuda et al. did not show a significant difference in average free T4 levels among GD patients with and without remission [10]. This might be the results from the difference in classification of remission and non-remission (active) stages between the current study and the previous study. Moreover, there were no other parameters associated with serum vitamin D levels in this study, including daily sun exposure, thyroid size, free T3, TSH, iPTH, and serum calcium level, which was in accordance with previous studies [13,17,18]. Although some factors such as daily sun exposure and calcium level should have an association with serum vitamin D levels, the impact of them might be too low to observe the correlation by statistical analysis.

The major limitation of this study was the small sample size. Because this study was performed during the COVID-19 pandemic, it was difficult to recruit many patients for the study. Another limitation was some confounding factors that cannot be eliminated, e.g., vitamin D supplements, vitamin D-rich food, and underestimation of sun exposure per day. These factors should be taken into account in future studies as suggested by the previous study [19]. Furthermore, further studies should focus more on the causation of vitamin D deficiency using a prospective methodology and control as many confounding factors as possible.

## 5. Conclusions

The patients with active GD had a lower vitamin D level compared to those with remission GD despite no statistical significance. In addition, the prevalence of vitamin D deficiency is significantly higher in the active GD group, and the patients with higher serum free T4 levels had lower serum vitamin D levels. Therefore, vitamin D levels should be monitored in patients with GD, especially in the active phase of GD.

## Figures and Tables

**Table 1:** Characteristics of patients with active and remission Graves’ disease (GD).

Characteristics	Active GD	Remission GD	*p*-Value
	(*n* = 41)	(*n* = 34)	
Age (year), mean (95% CI)	41.53 (31.46–51.60)	39.63 (32.29–46.96)	0.647
Male, *n* (%)	13 (31.71)	8 (23.53)	0.432
Weight (kg), mean (95% CI)	58.63 (55.12–62.15)	57.38 (51.18–63.57)	0.962
Height (cm), mean (95% CI)	163.07 (159.03–167.10)	158.88 (155.08–162.67)	0.072
Body mass index (kg/m^2^), mean (95%CI)	22.09 (20.75–23.44)	22.85 (20.13–25.56)	0.456
Goiter size (g), mean (95%CI)	55 (36.66–73.34)	31.56 (26.44–36.68)	<0.001
Current smoking, *n* (%)	8 (19.51)	4 (11.76)	0.362
Duration of disease (day), mean (95%CI)	834 (527–1142)	1454 (1086–1822)	0.003
Sun exposure (h/day), mean (95%CI)	3.53 (2.10–4.97)	3.08 (1.77–4.39)	0.781
Free T3 (pg/mL), mean (95%CI)	10.32 (6.77–13.88)	2.87 (2.45–3.28)	<0.001
Free T4 (ng/dL), mean (95%CI)	3.21 (2.39–4.02)	1.52 (1.13–1.91)	<0.001
TSH (mIU/L), mean (95% CI)	0.022 (0.008–0.035)	2.425 (0.756–4.094)	<0.001
iPTH (pg/mL), mean (95% CI)	45.81 (26.33–65.28)	38.97 (28.89–49.05)	0.543
Calcium (mg/mL), mean (95% CI)	9.25 (9.04–9.45)	9.35 (9.15–9.55)	0.417
Albumin (mg/mL), mean (95% CI)	4.19 (4.02–4.36)	4.48 (4.28–4.67)	<0.001

**Table 2:** Correlation coefficient (r) and *p*-value (P) of thyroid-related tests and vitamin D-related tests.

	Thyroid Size	Free T3	Free T4	TSH	iPTH	Calcium	Vitamin D Level
Daily sun exposure	r = 0.127	r = −0.092	r = −0.141	r = −0.194	r = 0.062	r = 0.062	r = 0.206
	*p* = 0.298	*p* = 0.493	*p* = 0.236	*p* = 0.172	*p* = 0.605	*p* = 0.599	*p* = 0.079
Thyroid size		r = 0.163	r = 0.131	r = −0.175	r = 0.102	r = 0.184	r = −0.073
		*p* = 0.245	*p* = 0.291	*p* = 0.239	*p* = 0.408	*p* = 0.131	*p* = 0.551
Free T3			r = 0.727 *	r = −0.717 *	r = −0.045	r = 0.037	r = −0.056
			*p* < 0.001	*p* < 0.001	*p* = 0.733	*p* = 0.783	*p* = 0.672
Free T4				r = −0.594 *	r = −0.020	r = −0.055	r = −0.254 *
				*p* < 0.001	*p* = 0.866	*p* = 0.648	*p* = 0.031
TSH					r = −0.078	r = 0.143	r = 0.159
					*p* = 0.586	*p* = 0.312	*p* = 0.260
iPTH						r = −0.281 *	r = −0.189
						*p* = 0.015	*p* = 0.108
Calcium							r = 0.182
							*p* = 0.118

* Statistical significance at *p*-value < 0.05.

## Data Availability

The data that support the findings of this study are available from the corresponding author upon reasonable request.
